# NF-κB: A Diverse and Multifunctional Transcription Factor in Holozoans

**DOI:** 10.1093/molbev/msag059

**Published:** 2026-03-09

**Authors:** Benjamin H Glass, Timinte Abraham, Trevor Siggers, Sarah W Davies, Thomas D Gilmore

**Affiliations:** Department of Biology, Boston University, Boston, MA 02215, USA; Department of Biology, Boston University, Boston, MA 02215, USA; Department of Biology, Boston University, Boston, MA 02215, USA; Department of Biology, Boston University, Boston, MA 02215, USA; Department of Biology, Boston University, Boston, MA 02215, USA

**Keywords:** NF-κB, evolution, immunity, symbiosis, development, signal transduction

## Abstract

Transcription factor nuclear factor-kappa B (NF-κB) and many upstream signaling components have been identified in a diversity of holozoan taxa, including unicellular holozoans (eg Filasterea and Choanoflagellata) and the metazoan phyla Porifera (sponges), Placozoa, and Cnidaria (eg jellyfishes, sea anemones, corals, and hydra). Herein, we review recent progress made toward characterizing the structure, regulation, activity, and biological functions of NF-κB proteins found in these taxa. We also provide an updated phylogenetic sampling of NF-κB orthologs highlighting their different domain configurations among holozoans, as well as a method for comparing the computationally predicted three-dimensional structures of NF-κB dimers and relating these structures to their amino acid similarities and DNA-binding specificities. This synthesis reveals new insights regarding the evolutionarily conserved and variable domain-dependent activities and regulation of holozoan NF-κBs. Further, we provide an overview of the roles of NF-κB in pathogen responses, stress responses, symbiosis, and development, with a focus on recent findings from sponges and cnidarians. This curation of a growing body of knowledge highlights both conserved and divergent roles of NF-κB in foundational biological processes. Finally, we suggest priorities for future research on the evolution of NF-κB structure and function. Overall, investigations of NF-κB in diverse holozoan taxa will continue to provide information about the origins of this important and pervasive transcriptional regulator and will also contribute to an understanding of the responses of sentinel species to the modern-day stresses associated with changing environmental conditions and novel pathogen-based diseases.

## Introduction

Nuclear factor-kappa B (NF-κB) transcription factors (TFs)—originally described for their roles in cancer (an avian retrovirus), embryonic development (*Drosophila melanogaster*), and B-cell immunity (mice)—are now known to be deeply evolutionarily conserved with roles in many foundational biological processes (Gilmore and Wolenski 2012; Williams and Gilmore 2020; Leger et al. 2022). Nevertheless, the vast majority of knowledge about these TFs still comes from a small number of taxa, namely, insects and vertebrates.

**Figure 1:**
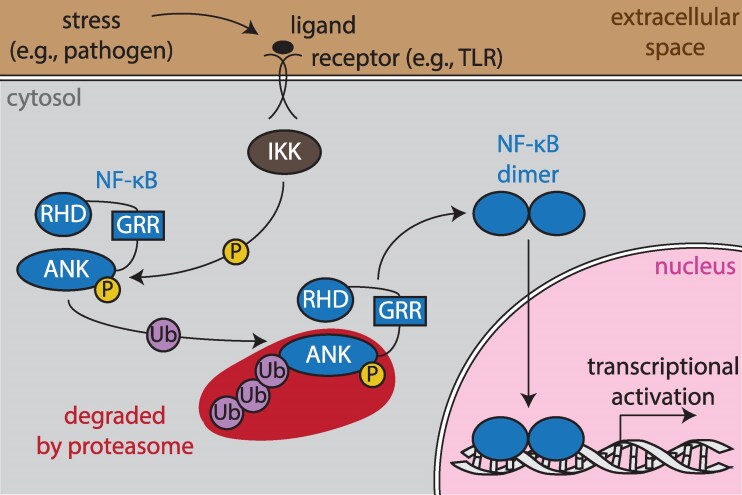
Simplified schematic of noncannonical NF-κB signaling (in mammals). In response to a stressor (eg a pathogen), cell surface receptors (eg Toll-like receptors [TLRs]) become activated and signal to IκB kinase (IKK; brown), which phosphorylates (P; yellow) conserved serine residues in the ankyrin (ANK) repeat of NF-κB (blue). This phosphorylation promotes ubiquitination (Ub; purple), resulting in the proteasomal degradation of NF-κB's autoinhibitory ANK domain up to the glycine-rich region (GRR). Finally, an NF-κB dimer enters the nucleus and binds to DNA (gray) via the Rel homology domain (RHD), resulting in the transcriptional activation of target genes for a given biological outcome.

In species from flies to humans, there are multiple NF-κB family proteins that are subdivided into the NF-κB and Rel proteins. Specifically, humans, mice, and most vertebrates have two NF-κB proteins (NF-κB1/p105 and NF-κB2/p100) and three Rel proteins (REL, RELA, and RELB) (Gilmore 2006). The vertebrate p105 and p100 proteins, and NF-κBs in other bilaterian taxa, have a conserved domain structure that includes an N-terminal Rel homology domain (RHD) that mediates dimerization, DNA binding, and nuclear localization, followed by a glycine-rich region (GRR) and series of inhibitory ankyrin (ANK) repeats (Gilmore 2006; Fig. 1). Mammalian NF-κB p105 and p100 are normally in an inactive state in the cytosol. In response to an upstream signal, a cluster of serine residues are phosphorylated by an IκB kinase (IKK), inducing proteasome-mediated removal of the ANK repeat sequences up to the GRR (Gilmore 2006; Fig. 1). The resulting shortened p50 and p52 NF-κB proteins can then localize to the nucleus and bind to κB sites in the promoters of target genes to activate their transcription (Gilmore 2006; Mankan et al. 2009; Fig. 1). In humans and mice, many of these target genes are involved in immunity, stress responses, and metabolism (Yang et al. 2016). The Rel proteins in metazoans are generally regulated in a second pathway, called the canonical pathway, that involves separate ANK repeat inhibitory IκB proteins (Gilmore 2006).

It is now clear that NF-κB proteins and upstream signaling pathways are pervasive across Holozoa, including unicellular holozoans (eg Filasterea and Choanoflagellata) and species in the metazoan phyla Porifera, Placozoa, and Cnidaria (Gilmore and Wolenski 2012; Williams and Gilmore 2020; Leger et al. 2022). In this review, we describe what is known, and can be inferred, from these NF-κB proteins in terms of their structures, regulation, and the biological processes influenced by them. Further, we have used computational modeling to compare the structures and DNA-binding properties of these NF-κB proteins.

Below, we first review the varied functional domain configurations of NF-κB now identified in a diversity of holozoan species and add to this summary new findings from several additional species for which this information has recently become available (see section “Functional domain configurations of NF-κB proteins in holozoans”). Next, we discuss the evolution of the NF-κB RHD, aided by a new analysis of RHD amino acid sequence conservation (see section “Sequence evolution of the NF-κB RHD”). Following this, we present a method for comparing RHD structures using the protein structure prediction program *AlphaFold3* and relate results from an example application of this method to a previously published characterization of NF-κB binding specificity (see section “Structural conservation of the NF-κB RHD and implications for DNA-binding activity”). We then review previous findings regarding the roles of NF-κB in various biological processes (see section “Roles of NF-κB in the biology of holozoans”), before finally discussing productive future research on this TF. Overall, this review aims to synthesize what is known about the structure and function of NF-κB across a diverse set of holozoan phyla, with new analyses of published data serving both to summarize previous findings and highlight how these findings, when viewed collectively and coupled with novel analytical tools, can reveal new insights about this important and widespread TF.

## Functional domain configurations of NF-κB proteins in holozoans

Based on comparative phylogenetic analyses, poriferans and cnidarians exclusively have NF-κB, but not Rel, proteins (Gilmore and Wolenski 2012; Williams and Gilmore 2020; Leger et al. 2022), leading to the hypothesis that the NF-κB subfamily first appeared prior to the divergence of Cnidaria and Bilateria (Gilmore and Wolenski 2012; Leger et al. 2022). The amino acid sequences and functional domain configurations of NF-κB proteins display considerable variation across holozoan taxa (Gilmore and Wolenski 2012; Williams and Gilmore 2020; Leger et al. 2022), suggesting substantial selection on the functions of these proteins and their regulation. In contrast to bilaterians, most poriferans and cnidarians have single NF-κB proteins rather than multiple paralogs, indicating that NF-κB homodimers are the primary effector TFs of this family in these organisms (Gilmore and Wolenski 2012; Williams and Gilmore 2020). However, some choanoflagellates have multiple NF-κB-like proteins (Williams et al. 2021).

**Figure 2:**
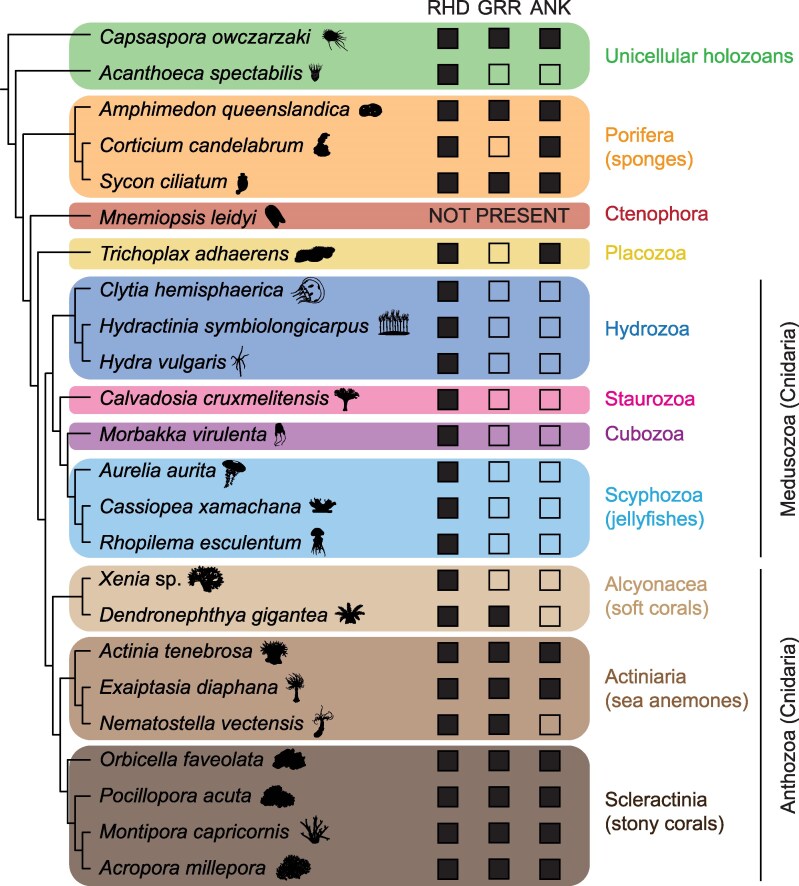
Domain configurations of NF-κB orthologs among holozoans. Phylogenetic tree of hypothesized relationships among taxa and domain content of NF-κB orthologs from representative species. Major domains identified include the Rel homology domain (RHD), glycine-rich region (GRR), and ankyrin repeat region (ANK). Filled boxes indicate the presence of (known or predicted) domains in the depicted NF-κB orthologs. Certain positions of taxa depicted in this tree are still debated, including Porifera as the sister taxon to all other metazoan phyla (Najle et al. 2023; Schultz et al. 2023), and Hydrozoa as the sister class to Staurozoa, Cubozoa, and Scyphozoa within Medusozoa (Zapata et al. 2015; Feng et al. 2023). Organism silhouettes were sourced from phylopic.org.

Across the unicellular holozoans, Porifera, Placozoa, and Cnidaria, there are at least four NF-κB protein configurations that differ in domains and sequences outside the RHD: (i) NF-κB proteins with structures similar to vertebrate NF-κBs, with GRRs, ANK repeats, and signal-induced phosphorylation sites; (ii) proteins with an ANK repeat domain but no GRR or phosphorylation sites; (iii) NF-κBs with the GRR but no ANK repeat domain; and (iv) C-terminally truncated proteins retaining only the RHD (Fig. 2). Of note, no NF-κB ortholog has yet been identified in a species belonging to the phylum Ctenophora (Fig. 2), suggesting that this TF was lost during the evolution of this phylum.

### Unicellular holozoans

The NF-κB ortholog from the filasterean *Capsaspora owczarzaki* displays a domain structure akin to that of vertebrate NF-κBs, including the RHD, GRR, and ANK domains (Fig. 2), yet there is low amino acid sequence conservation (<30%) between this protein and orthologs from insects and vertebrates (Richter et al. 2018; Williams et al. 2021). Despite this divergence, the *C. owczarzaki* NF-κB displays properties that are similar to vertebrate NF-κBs when expressed in a human cell line (HEK 293T). Specifically, the full-length *C. owczarzaki* NF-κB is located in the cytoplasm, while a truncated version of this protein containing only the N-terminal RHD—simulating the proteasome-mediated processing of human NF-κB p100 and 105—can enter the nucleus and activate transcription (Williams et al. 2021). In contrast, three NF-κB orthologs from the choanoflagellate *Acanthoeca spectabilis* have only the RHD (Fig. 2), and, when expressed in vertebrate cells in vitro, are present in the nucleus as dimers and are able to activate transcription (Williams et al. 2021). These findings demonstrate a deeply conserved role of the RHD in nuclear translocation and transcriptional activation. However, there are some choanoflagellate species with no known NF-κB genes/proteins (Williams et al. 2021).

### Porifera, Placozoa, and Cnidaria

The NF-κB protein in the model sponge *Amphimedon queenslandica* displays an extended RHD-GRR-ANK domain structure and similar regulation to the *C. owczarzaki* and human NF-κBs (Gauthier and Degnan 2008) (Fig. 2), with proteasome-mediated C-terminal truncation inducing nuclear translocation and subsequent transcriptional activation (Williams et al. 2020). However, the sponge NF-κB protein also has serines that can serve as IKK phosphorylation sites (Williams et al. 2020). This suggests that regulation of NF-κB activity by IKK arose early in metazoan evolution.

NF-κB proteins in placozoans are less studied than those in other nonmodel taxa. However, a purported NF-κB ortholog was identified in the genome of the placozoan *Trichoplax adhaerens* (Kamm et al. 2019). This protein has predicted structural similarity to human p100, possessing a highly derived RHD and ANK repeats but lacking a GRR (Kamm et al. 2019; Romanova and Moroz 2025) (Fig. 2). This suggests that at least some placozoan species either retained or convergently evolved NF-κB TFs.

Among the cnidarians, the hydrozoans *Hydra vulgaris* and *Clytia hemisphaerica*, and the scyphozoan jellyfish *Aurelia aurita*, have NF-κB proteins that, like that in the choanoflagellate *A. spectabilis*, contain only RHDs (Williams and Gilmore 2020; Williams et al. 2025) (Fig. 2). As with *A. spectabilis* NF-κB and processed human p50 and p52, the orthologs from *C. hemisphaerica* and *A. aurita* localize to the nucleus and have the ability to activate transcription (in vertebrate cells) (Williams et al. 2025). This further supports the hypothesis that the RHD is sufficient for nuclear translocation and transcriptional activation across a diversity of holozoans.

In contrast to those medusozoans, the anthozoan sea anemones *Exaiptasia diaphana* and *Actinia tenebrosa* (Actiniaria) have NF-κB orthologs that are similar to vertebrate NF-κB p100, with the extended RHD-GRR-ANK-phosphorylation site structure (Mansfield et al. 2017; Emery et al. 2021) (Fig. 2). In accordance with findings from *C. owczarzaki* and humans, the full-length *E. diaphana* NF-κB is localized to the cytoplasm, while a C-terminally truncated version can enter the nucleus and activate transcription in cell lines (Mansfield et al. 2017). Interestingly, an *E. diaphana* IKK homolog can induce proteasome-dependent processing of this species' NF-κB when co-expressed in human cells (Mansfield et al. 2017), further supporting the hypothesis that IKK-dependent regulation arose early in the evolution of metazoans. In contrast, the closely related sea anemone *Nematostella vectensis* has an NF-κB ortholog with an RHD-only structure that is similar to the NF-κBs of *A. spectabilis*, *C. hemisphaerica*, and *H. vulgaris*, having lost the ANK domain (Sullivan et al. 2007, 2009; Wolenski et al. 2011a; Mansfield et al. 2017) (Fig. 2). As with its close orthologs, NF-κB of *N. vectensis* displays nuclear localization and transcriptional activation capacity when expressed in cell lines (Wolenski et al. 2011a; Ryzhakov et al. 2013; Mansfield et al. 2017). Interestingly, the *N. vectensis* NF-κB also contains a GRR, which is likely a remnant of a gene splitting event that generated an independent IκB gene (Gilmore and Wolenski 2012).

The RHD-only configuration is also predicted for the NF-κB orthologs from the staurozoan *Calvadosia cruxmelitensis*, cubozoan *Morbakka virulenta*, scyphozoan *Cassiopea xamachana*, and soft corals (Alcyonacea) *Xenia* sp. and *Dendronephthya gigantea* (Emery et al. 2021) (Fig. 2). In contrast, the scleractinian coral *Orbicella faveolata*'s NF-κB is analogous to that of *E. diaphana* and humans, with an RHD-GRR-ANK domain structure and nuclear localization/activity in cell lines only when C-terminally truncated (Williams et al. 2018) (Fig. 2). This extended domain structure is also observed in the predicted orthologs from the corals *Pocillopora acuta*, *Acropora millepora*, and *Montipora capricornis* (Emery et al. 2021) (Fig. 2). These findings from Cnidaria highlight that NF-κB's domain structure is highly variable between even species of the same cnidarian class.

Overall, findings from Porifera and Cnidaria are congruent with those from both unicellular holozoans and bilaterian taxa, specifically that NF-κBs containing ANK repeats are inactive in the cytoplasm when expressed in cell lines, whereas truncated proteins with RHDs are typically nuclear and can bind DNA to activate transcription. Thus, while NF-κB proteins across taxa display divergent amino acid sequences, the domain-dependent subcellular regulation of these proteins appears to be conserved across a broad diversity of taxa.

### An updated phylogenetic sampling of NF-κB domain configurations

As summarized above, the structures of NF-κB proteins from a diverse set of holozoan species have now been described, and rules for conserved domain-dependent regulation are evident. Here, we describe the domain architecture of NF-κB proteins from several additional poriferans and cnidarians. First, the sponge *Sycon ciliatum* has one predicted NF-κB protein with an RHD-GRR-ANK domain structure, similar to the closely related sponge *A. queenslandica* (Fig. 2). Notably, the predicted NF-κB protein from the sponge *Corticium candelabrum* also contains RHD and ANK domains but lacks the GRR (Fig. 2). The scyphozoan *Rhopilema esculentum*, like the closely related jellyfish *A. aurita*, has a predicted NF-κB protein with only an RHD (Fig. 2). As described above, this RHD-only domain structure is also observed in the hydrozoans *C. hemisphaerica* and *H. vulgaris*, and we now add the hydrozoan *Hydractinia symbiolongicarpus* to this group (Fig. 2). Thus, NF-κBs from all of the staurozoans (one species), cubozoans (one species), scyphozoans (three species), and hydrozoans (three species) in which predicted proteins have been identified share the RHD-only domain configuration (Fig. 2). Notably, the soft corals *Xenia* sp. and *Dendronephythya gigantea* have predicted NF-κBs that also lack ANK repeats, making them similar to those of the hydrozoans and scyphozoans (Emery et al. 2021). However, *D. gigantea* is unique among this group in that its predicted NF-κB protein also contains a GRR—a domain configuration which, among the 23 species analyzed, is shared only with *N. vectensis* (Fig. 2). While this configuration may be shared with other *Dendronephthya*—a diverse genus with over 250 species (WoRMS Editorial Board 2025)—only *D. gigantea* has a high-quality genome available. Since NF-κBs from all of the stony corals and most of the sea anemones for which data are currently available display RHD-GRR-ANK domain structures (Fig. 2), these findings suggest that the RHD-only proteins observed in the medusozoans arose in this clade's ancestral lineage following the divergence from Anthozoa.

## Sequence evolution of the NF-κB RHD

**Figure 3:**
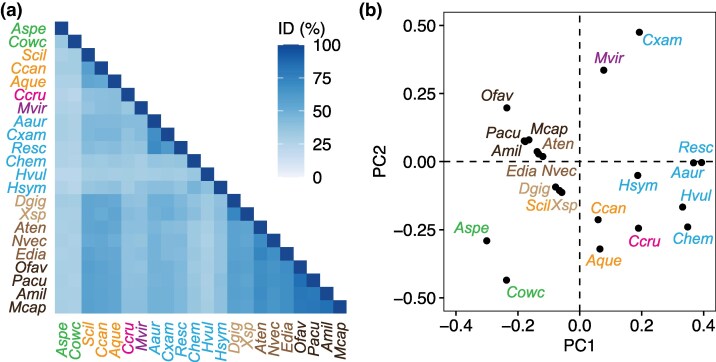
Amino acid sequence evolution of the NF-κB RHD. a) Percentage of amino acid identities (ID %) between the Rel homology domains (RHDs) of diverse NF-κBs from pairwise sequence alignments. b) RHDs by species, visualized along the first and second principal components (PCs) from a PCA of sequence alignment substitution scores (BLOSUM62). In both panels, text is colored by phylogenetic group as in Fig. 2: green, unicellular holozoans; orange, sponges; pink, staurozoan; purple, cubozoan; light blue, scyphozoans; dark blue, hydrozoans; beige, soft corals; light brown, sea anemones; dark brown, stony corals. Species abbreviations are as follows: *Aspe*, *Acanthoeca spectabilis*; *Cowc*, *Capsaspora owczarzaki*; *Scil*, *Sycon ciliatum*; *Ccan*, *Corcitium candelabrum*; *Aque*, *Amphimedon queenslandica*; *Ccru*, *Calvadosia cruxmelitensis*; *Mvir*, *Morbakka virulenta*; *Aaur*, *Aurelia aurita*; *Cxam*, *Cassiopea xamachana*; *Resc*, *Rhopilema esculentum*; *Chem*, *Clytia hemisphaerica*; *Hvul*, *Hydra vulgaris*; *Hsym*, *Hydractinia symbiolongicarpus*; *Dgig*, *Dendronephthya gigantea*; *Xsp*, *Xenia* sp.; *Aten*, *Actinia tenebrosa*; *Nvec*, *Nematostella vectensis*; *Edia*, *Exaiptasia diaphana*; *Ofav*, *Orbicella faveolata*; *Pacu*, *P. acuta*; *Amil*, *Acropora millepora*; *Mcap*, *Montipora capricornis*.

The recent characterization of NF-κB sequences from an expanded set of holozoan taxa offers the opportunity to investigate the sequence evolution of the RHD, which is central to NF-κB's function as a TF. The RHD contains a DNA-binding motif (R*RY) that is conserved across all currently identified orthologs, while other features including the dimerization domain and nuclear localization sequence (NLS) display more variation in amino acid sequence (Williams and Gilmore 2020; Leger et al. 2022). Interestingly, this variation is observed both across and within clades, and does not follow a strict phylogenetic pattern (Williams and Gilmore 2020; Leger et al. 2022). To illustrate this, we performed a multiple sequence alignment (MSA) of NF-κB RHDs from 22 species using *Clustal Omega* (Sievers and Higgins 2014; default settings, BLOSUM62 scoring matrix) and used this MSA to determine percent amino acid identities for each pair of species in addition to performing a principal component analysis (PCA) on pairwise similarity scores using *Jalview* (Waterhouse et al. 2009; Fig. 3). This analysis included all species depicted in Fig. 2 aside from the placozoan *T. adhaerens*, as this species' RHD is so highly derived as to preclude confident identification of its exact sequence boundaries. This analysis showed that the RHD of NF-κB from the sponge *S. ciliatum* displays higher amino acid sequence similarity to the RHDs of sea anemone and coral NF-κBs than with those of other sponges (Fig. 3a). Accordingly, the *S. ciliatum* RHD sequence clusters with those from *A. tenebrosa*, *E. diaphana*, and *N. vectensis* in the PCA (Fig. 3b). Additionally, the NF-κBs of the sponges *S. ciliatum*, *A. queenslandica*, and *C. candelabrum* display greater sequence similarity to NF-κBs of sea anemones and soft corals than is observed between these cnidarians and the more closely related *M. virulenta* and *C. cruxmelitensis* (Fig. 3a to b). The cnidarian *H. vulgaris* also presents a unique case of RHD sequence, displaying comparatively low levels of sequence conservation with all other species analyzed (Fig. 3a).

The selective factors driving the evolution of these diverse RHDs are currently unclear, and it is likely that a number of ecological and life history traits have shaped these patterns. Indeed, traits including symbiosis and coloniality were recently shown to be associated with the evolution of pattern recognition receptors and downstream pathway components in cnidarians (Emery et al. 2021), suggesting that the same could be true for NF-κB.

## Structural conservation of the NF-κB RHD and implications for DNA-binding activity

### Modeling conformations of NF-κB homodimers with *AlphaFold3*

**Figure 4:**
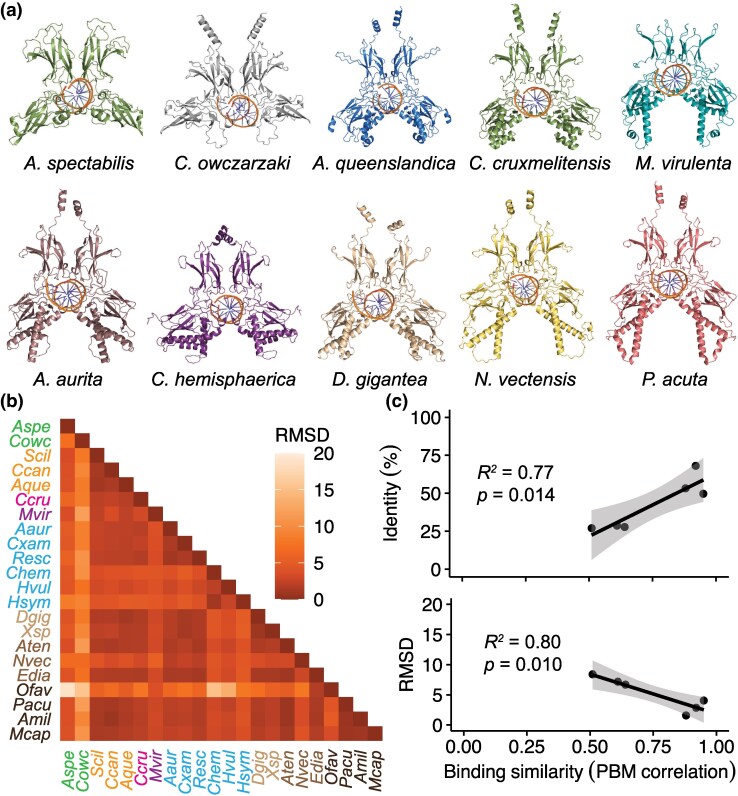
Conformational evolution of NF-κB orthologs and correlation with DNA-binding preference. a) Predicted 3D conformations of the indicated NF-κB RHD dimers bound to a double-stranded palindromic DNA sequence (5′-GGGTTAACCC-3′). b) Root mean square deviation (RMSD) between 3D structures of diverse dimers. Text is colored by phylogenetic group as in Fig. 2: green, unicellular holozoans; orange, sponges; pink, staurozoan; purple, cubozoan; light blue, scyphozoans; dark blue, hydrozoans; beige, soft corals; light brown, sea anemones; dark brown, stony corals. Species abbreviations are as follows: *Aspe*, *Acanthoeca spectabilis*; *Cowc*, *Capsaspora owczarzaki*; *Scil*, *Sycon ciliatum*; *Ccan*, *Corcitium candelabrum*; *Aque*, *Amphimedon queenslandica*; *Ccru*, *Calvadosia cruxmelitensis*; *Mvir*, *Morbakka virulenta*; *Aaur*, *Aurelia aurita*; *Cxam*, *Cassiopea xamachana*; *Resc*, *Rhopilema esculentum*; *Chem*, *Clytia hemisphaerica*; *Hvul*, *Hydra vulgaris*; *Hsym*, *Hydractinia symbiolongicarpus*; *Dgig*, *Dendronephthya gigantea*; *Xsp*, *Xenia* sp.; *Aten*, *Actinia tenebrosa*; *Nvec*, *Nematostella vectensis*; *Edia*, *Exaiptasia diaphana*; *Ofav*, *Orbicella faveolata*; *Pacu*, *Pocillopora acuta*; *Amil*, *Acropora millepora*; *Mcap*, *Montipora capricornis*. c) Correlations between amino acid identity percentage (top plot) or RMSD (bottom plot) and *z*-score correlation from protein-binding microarray (PBM) data originally from Mansfield et al. (2017), with corresponding *R*^2^ and *P*-values.

To complement analyses of the domain configurations and RHD amino acid sequences of NF-κB proteins, we leveraged recent advances in protein three-dimensional (3D) structural prediction to assess conservation of the NF-κB DNA-binding fold using *AlphaFold3* (Abramson et al. 2024). For structural modeling, we selected the NF-κB orthologs from 10 representative species spanning taxa from unicellular holozoans to cnidarians and included NF-κBs with a range of domain architectures (Fig. 2). For each NF-κB, we modeled RHD homodimers bound to a consensus, palindromic double-stranded DNA target sequence (5′-GGGTTAACCC-3′ and its reverse complement) in order to compare structural features of dimerization and DNA interaction (Fig. 4a). Modeled homodimers demonstrate both conserved and divergent predicted conformational features, which are not solely explained by phylogeny (Fig. 4a). For example, most of the homodimers contain a pair of predicted α-helices C-terminal to the DNA-binding fold; however, these structures are absent in the homodimers from *A. spectabilis* and *M. virulenta* (Fig. 4a, superior to binding folds). Also notable is the interspecific variation in the number of predicted α-helices that are proximal to the DNA-binding fold, with none of these helices present in the *C. owczarzaki* structure, two helices per monomer present for *A. spectabilis*, and three per monomer (typically two longer and one shorter) present for all other species (Fig. 4a, inferior to binding folds). These results suggest that multiple helices arose in this structural domain in the common ancestor of Porifera and Cnidaria, with these structures having been retained over time. However, additional analyses incorporating data from a broader diversity of taxa are needed to more fully reconstruct the full evolution of NF-κB structure.

Structural similarity between *AlphaFold3*-predicted structures was calculated as the root mean square deviation (RMSD) of the backbone C_ɑ_ atoms following structural alignment using *PyMOL* (Yuan et al. 2017) and provides additional insights into evolution of protein structure. Interestingly, RMSD values generally reflect phylogenetic relationships, with low RMSD between predicted homodimers from species that are more closely related by sequence-based phylogenetic analysis, although there are some exceptions (Fig. 4b). One notable exception is the predicted homodimer from the coral *O. faveolata*, which displays a relatively high RMSD as compared to the NF-κB homodimers of all other species in this dataset, including other stony corals (Fig. 4b). This difference in the structure of *O. faveolata*'s NF-κB may be largely explained by the presence of a unique 38-amino acid sequence near the middle of this species' RHD (Williams et al. 2018). Overall, as protein conformation prediction software continues to improve, structure-based analyses will become increasingly insightful and important for determining ancestral and derived characteristics of key transcription factors, as well as other proteins, in nonmodel organisms.

### Relationship between RHD sequence, structure, and DNA-binding specificity

The innovative application of protein-binding microarray (PBM) technology to NF-κB proteins from *C. owczarzaki*, *A. queenslandica*, *N. vectensis*, and *E. diaphana* showed that these proteins display a high level of similarity in DNA-binding site specificity (Mansfield et al. 2017). However, there is still some variation in specificity (expressed as the correlation of PBM *z*-scores for over 2,600 possible κB sites) for pairs of these NF-κB orthologs. New sequence and structural information for these NF-κB proteins allows for a comparison of relationships between sequence similarity, RMSD-based structure, and DNA-binding site specificity. Here, we correlated both amino acid sequence similarity and pairwise RMSD values with DNA-binding site preference similarity (ie correlation of PBM *z*-scores) and analyzed these correlations via linear regression (Fig. 4c). Interestingly, both amino acid sequence similarity (percent identities) and pairwise RMSD values display strong (*R*^2^ > 0.75), statistically significant (*P* < 0.05) correlations with DNA-binding similarity (Fig. 4c). These results add nuance to the previous hypothesis that there has not been substantial evolution of the DNA-binding site preference of NF-κB proteins (Mansfield et al. 2017), and suggest that there are RHD sequence features under selection, which have influenced DNA-binding site specificity over the evolutionary history of this TF.

## Roles of NF-κB in the biology of holozoans

As our knowledge of the structure, function, and regulation of NF-κB in holozoans has grown, so too has our understanding of the biological roles of this pervasive TF. It is becoming clear that NF-κB plays roles in pathogen responses, symbioses, development, and stress responses in a diversity of taxa (Table 1), as reviewed below.

**Table 1:** Biological roles of NF-κB in holozoans from Filasterea, Porifera, and Cnidaria.

Species	Taxon	Apparent role	NF-κB or upstream	Condition	Response	Reference
*Aplysina aerophoba*	Porifera	Pathogen response	Upstream	Microbial-origin small molecule exposure	Increased expression (mRNA)	Pita et al. (2018)
*Dysidea avara*	Porifera	Pathogen response	Upstream	Microbial-origin small molecule exposure	Increased expression (mRNA)	Pita et al. (2018)
*Cassiopea xamachana*	Cnidaria, Scyphozoa	Pathogen response	NF-κB	Pathogen exposure	Increased expression (mRNA)	Emery et al. (2024)
*Orbicella faveolata*	Cnidaria, Scleractinia	Pathogen response	NF-κB	Pathogen exposure	Increased expression (mRNA)	Fuess et al. (2017)
*Nematostella vectensis*	Cnidaria, Actiniaria	Pathogen response	Upstream	Pathogen exposure	Increased expression (mRNA)	Brennan et al. (2017); Carrión et al. (2023)
*Nematostella vectensis*	Cnidaria, Actiniaria	Pathogen response	NF-κB	Knockdown + microbial-origin small molecule exposure	Lack of proper innate immune response	Margolis et al. (2021)
*Nematostella vectensis*	Cnidaria, Actiniaria	Pathogen response	NF-κB	Viral-origin small molecular exposure	Increased expression (mRNA)	Kozlovski et al. (2025)
*Neopetrosia compacta*	Porifera	Stress response	NF-κB	Heat	Increased expression (mRNA)	Posadas et al. (2022)
*Nematostella vectensis*	Cnidaria, Actiniaria	Stress response	NF-κB	Starvation	Decreased expression (mRNA and protein)	Carrión et al. (2023)
*Exaiptasia diaphana*	Cnidaria, Actiniaria	Stress response	NF-κB	Starvation and heat	Increased expression (mRNA and protein)	Cleves et al. (2020); Valadez-Ingersoll et al. (2024); Sharoni et al. (2025)
*Exaiptasia diaphana*	Cnidaria, Actiniaria	Stress response	NF-κB	Hosting of heterologous algae	Increased expression (protein)	Mansfield et al. (2019)
*Nematostella vectensis*	Cnidaria, Actiniaria	Stress response	NF-κB	Heat	Increased expression (mRNA)	Sharoni et al. (2025)
*Astroides calycularis*	Cnidaria, Scleractinia	Stress response	NF-κB	Heat	Increased expression (mRNA)	Bisanti et al. (2024)
*Acropora palmata*	Cnidaria, Scleractinia	Stress response	NF-κB	Heat	Increased expression (mRNA)	DeSalvo et al. (2010)
*Acropora hyacinthus*	Cnidaria, Scleractinia	Stress response	NF-κB	Heat	Increased expression (mRNA)	Traylor-Knowles et al. (2017)
*Stylophora pistillata*	Cnidaria, Scleractinia	Stress Response	NF-κB	Heat	Increased expression (mRNA)	Sharoni et al. (2025)
*Ephydatia muelleri*	Porifera	Symbiosis	Upstream	Active symbiosis	Decreased expression (mRNA)	Hall et al. (2021)
*Cassiopea xamachana*	Cnidaria, Scyphozoa	Symbiosis	NF-κB	Active symbiosis	Decreased expression (mRNA)	Emery et al. (2021)
*Exaiptasia diaphana*	Cnidaria, Actiniaria	Symbiosis	NF-κB	Active symbiosis; microbiome depletion	Decreased expression (mRNA and protein)	Mansfield et al. (2017); Valadez-Ingersoll et al. (2024); Valadez-Ingersoll et al. (2026)
*Oculina arbuscula*	Cnidaria, Scleractinia	Symbiosis	NF-κB	Active symbiosis	Decreased expression (mRNA)	Rivera and Davies (2021); Valadez-Ingersoll et al. (2025)
*Capsaspora owczarzaki*	Filasterea	Differentiation	NF-κB	Life-stage transition	Variable expression (protein)	Williams et al. (2021)
*Amphimedon queenslandica*	Porifera	Development	NF-κB	Larval development	Increased expression (mRNA)	Gauthier and Degnan (2008)
*Clytia hemisphaerica*	Cnidaria, Hydrozoa	Development	NF-κB	Larval development	Increased expression (mRNA)	Williams et al. (2025)
*Nematostella vectensis*	Cnidaria, Actiniaria	Development	NF-κB	Larval development	Increased expression (mRNA)	Warner et al. (2018); Cole et al. (2024)
*Nematostella vectensis*	Cnidaria, Actiniaria	Development	Upstream (TLR)	Knockdown via morpholino	Severe developmental abnormalities	Brennan et al. (2017)
*Acropora . millepora*	Cnidaria, Scleractinia	Development	NF-κB	Exposure to settlement cue	Increased expression (mRNA)	Siboni et al. (2014)

### Pathogen responses

Among the most well-characterized roles of NF-κB in bilaterians is its role in pathogen responses as a critical effector TF for innate immunity (Dev et al. 2011; Gilmore and Wolenski 2012). Growing evidence suggests that a role for NF-κB in pathogen response is conserved in some sponges and cnidarians (Table 1). For example, the sponges *Aplysina aerophoba* and *Dysidea avara* show increases in the expression of genes involved in NF-κB signaling upon exposure to microbial small molecules (Pita et al. 2018). Further, pathogen infection leads to an increase in NF-κB mRNA expression in the scyphozoan *C. xamachana* (Emery et al. 2024) and activates NF-κB signaling in the coral *O. faveolata*, although this response is notably absent in the corals *Pseudodiploria strigosa*, *Porites porites*, and *Porites astreoides* (Fuess et al. 2017). Simulated or actual pathogen exposure also activates NF-κB signaling in the sea anemone *N. vectensis* (Brennan et al. 2017; Carrión et al. 2023; Kozlovski et al. 2025), and shRNA-mediated knockdown of NF-κB in this species prevents the proper activation of antibacterial genes in response to a bacterial small molecule (Margolis et al. 2021). Similarly, transgenic *H. vulgaris* with drastically reduced expression of myeloid differentiation primary response gene 88 (MyD88), a key adapter protein for activation of NF-κB, are more susceptible to bacterial infection than control animals (Franzenburg et al. 2012). These findings suggest that NF-κB plays a role in activating an innate immune response in sponges and cnidarians, a function apparently conserved across several species.

### Stress responses

The role of NF-κB in organismal responses to (nonpathogen) stressors is often less emphasized compared to its functions in immunity; however, it has become clear that diverse stressors modulate NF-κB signaling in many holozoans (Table 1). For example, NF-κB is upregulated at the mRNA level under heat stress in the sponge *Neopetrosia compacta* (Posadas et al. 2022), the cnidarians *Astroides calycularis* (Bisanti et al. 2024), *Acropora palmata* (DeSalvo et al. 2010), *Acropora hyacinthus* (Traylor-Knowles et al. 2017), *Stylophora pistillata* (Sharoni et al. 2025), *E. diaphana* (Mansfield et al. 2017; Cleves et al. 2020; Sharoni et al. 2025), and *N. vectensis* (Sharoni et al. 2025), as well as likely in many other species. We hypothesize that NF-κB is increased under heat stress in these organisms to regulate cellular processes that promote stress acclimation, such as the clearing of misfolded proteins and damaged organelles and antioxidant responses. Indeed, in *E. diaphana*, genes highly upregulated under heat stress display an enrichment of predicted NF-κB binding sites in their proximal promoter regions, and these genes are implicated in processes including proteolysis (eg multiple E3 protein-ubiquitin ligases) (Cleves et al. 2020).

However, in *S. pistillata*, *E. diaphana*, and *N. vectensis*, not all individuals exposed to heat stress upregulate NF-κB (Sharoni et al. 2025). In particular, corals originating from habitats experiencing extreme heat and/or high levels of thermal variability (eg the Red Sea) do not show upregulation of NF-κB in response to heat stress (Sharoni et al. 2025), potentially indicating that NF-κB is constitutively upregulated (“frontloaded”; Barshis et al. 2013) in these organisms and/or that other mechanisms not related to NF-κB are used to promote heat tolerance. These population-specific responses may be the result of acclimatization or selection on the NF-κB gene itself, the latter of which has been observed in *N. vectensis*, with populations along a latitudinal cline exhibiting two alleles of NF-κB that have distinct DNA-binding activities (Sullivan et al. 2009; Wolenski et al. 2011b).

Expression of NF-κB is similarly upregulated in the sea anemone *N. vectensis* following exposure to heavy metal stress (Elran et al. 2014), and in the closely related *E. diaphana* upon introduction of heterologous algae and starvation (Mansfield et al. 2019). On the other hand, starvation has contrasting effects on NF-κB levels in *N. vectensis* and *E. diaphana*, with this stressor leading to a decrease in NF-κB mRNA and protein expression in *N. vectensis* (Carrión et al. 2023), but an increase in expression in *E. diaphana* (Valadez-Ingersoll et al. 2024). As observed under heat stress, the upregulation of NF-κB under starvation in *E. diaphana* is associated with the activation of purported target genes involved in processes that may promote survival, such as inflammation and the response to reactive oxygen species (Valadez-Ingersoll et al. 2024). It is unclear why this response is not observed in *N. vectensis*, particularly as predicted NF-κB target genes in this species include many that are also predicted for *E. diaphana*, such as *BCL3* and multiple tumor necrosis factor receptor-associated factor 3-like (TRAF) genes (Wolenski et al. 2011a; Margolis et al. 2021; Carrión et al. 2023). Nonetheless, it is clear that NF-κB is modulated by a diverse array of stressors, suggesting its importance in coordinating acclimatory gene expression to promote persistence under adverse conditions.

### Symbiosis

In diverse symbioses from the legume-rhizobia partnership to the human gut microbiome, interactions between microbes and their hosts often involve suppression of the host immune system to allow for tolerance of the symbiont (Gross et al. 2009). Mounting evidence suggests that the suppression of NF-κB activity through the modulation of NF-κB protein itself and/or upstream signaling components is a mechanism by which symbioses are established and maintained in sponges and cnidarians (Table 1). First, when compared to aposymbiotic individuals lacking *Chlorella*-like symbionts, individuals of the freshwater sponge *Ephydatia muelleri* possessing symbionts display upregulated expression of multiple TRAFs, which are typically negative regulators of NF-κB activity (Hall et al. 2021). Similarly, among cnidarians, NF-κB mRNA and/or protein levels are decreased in symbiotic compared to aposymbiotic states in the scyphozoan *C. xamachana* (Emery et al. 2021) and anthozoans *E. diaphana* (Mansfield et al. 2017; Weizman and Levy 2019; Valadez-Ingersoll et al. 2024) and *Oculina arbuscula* (Rivera and Davies 2021; Valadez-Ingersoll et al. 2025). Recently, NF-κB protein levels have also been shown to be decreased in *E. diaphana* following microbiome depletion (Valadez-Ingersoll et al. 2026). These findings are consistent with those from symbioses in other organisms, including the salamander-green alga symbiosis, in which algal establishment inhibits NF-κB (Burns et al. 2017).

Based on these findings, NF-κB appears to be a broadly conserved protein target for modulation in symbiosis. Notably, a recent single-cell RNA sequencing dataset from *O. arbuscula* revealed the downregulation of multiple purported NF-κB pathway genes specifically in the symbiont-hosting gastrodermal cells, with no differences in the expression of the same genes in symbiont-free immune cells between aposymbiotic and symbiotic animals (Valadez-Ingersoll et al. 2025). These results are in agreement with those from larvae of *E. diaphana*, in which the expression of immune genes, including some purported NF-κB pathway genes, was found to be downregulated specifically in symbiont-hosting cells (Jacobovitz et al. 2021). It is worth noting, however, that the NF-κB gene itself was upregulated in symbiont-hosting cells of *E. diaphana* larvae (Jacobovitz et al. 2021), leading to some ambiguity as to how the activity of NF-κB is modulated under symbiosis in this context. Regardless, cell- and tissue-specific results from *O. arbuscula* and *E. diaphana* illustrate the complexity of NF-κB regulation in cnidarian symbioses and highlight the value of coupling both whole organism and cell type-specific data in understanding the molecular and cellular mechanisms underlying host–symbiont relationships.

### Development

The importance of NF-κB in early (eg embryonic) development is well-established in bilaterian species including *D. melanogaster* and mammals, with NF-κB playing key roles in dorsal–ventral patterning, notochord formation, limb and liver development, and hematopoiesis (Espín-Palazón and Traver 2016). NF-κB is also likely involved in life-stage transitions in *C. owczarzaki* and development in some sponges and cnidarians (Table 1). In *C. owczarzaki*, NF-κB protein activity varies across life stages (Williams et al. 2021). In the sponge *A. queenslandica* NF-κB is expressed during early development, with cell type-specific expression patterns in larvae (Gauthier and Degnan 2008). Further, in the jellyfish *C. hemisphaerica*, the NF-κB gene shows a rapid increase in expression following fertilization, peaking during the early gastrula and planula stages before decreasing in primary polyps and remaining comparatively low in adults (Williams et al. 2025). Interestingly, similar expression patterns for both NF-κB and IκB mRNA and protein are observed in the anthozoan *N. vectensis* (Warner et al. 2018; Cole et al. 2024), and blocking translation of the upstream Toll-like receptor (TLR) via morpholino injection in zygotes results in severe developmental abnormalities at day 5 postfertilization (Brennan et al. 2017). These results, however, are complicated by the finding that morpholino-mediated disruption of NF-κB expression in *N. vectensis* zygotes abolishes the formation of pre-cnidocyte stinging cells, but does not lead to any overt early developmental defect (Wolenski et al. 2013). Thus, the precise role of NF-κB and its interaction with TLR signaling in *N. vectensis* development remains unclear. The role of NF-κB in coral development is also unknown; however, exposure to a metamorphosis-inducing cue leads to increased NF-κB mRNA expression in larvae of the coral *A. millepora* (Siboni et al. 2014). Together, these findings suggest a role for NF-κB in life-stage transitions in some unicellular holozoans and development in sponges and cnidarians.

## Conclusions and future directions

**Figure 5:**
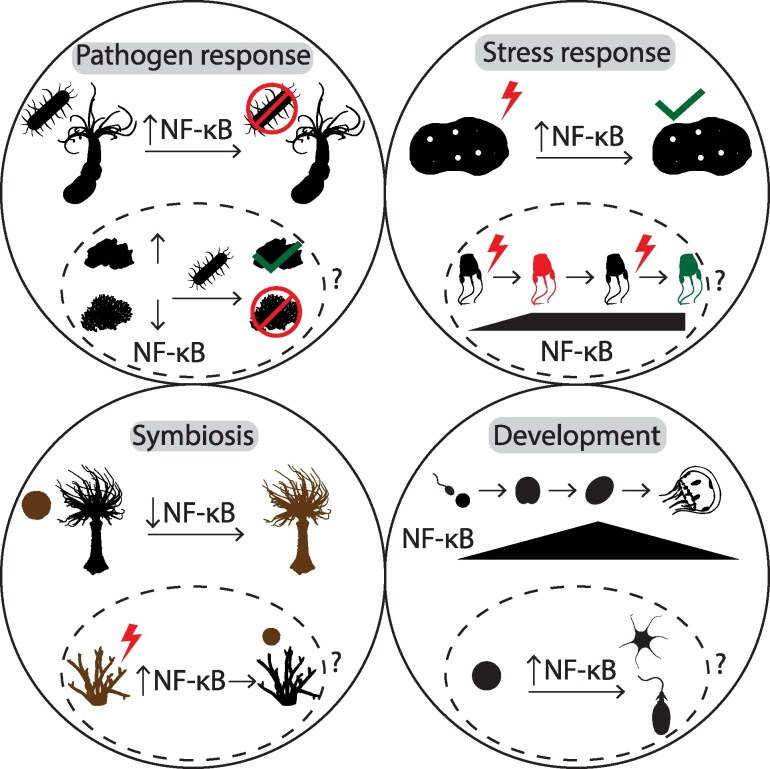
Established and hypothesized biological roles of NF-κB. There is now evidence for roles of NF-κB in pathogen responses, stress responses, symbiosis, and development in a diversity of holozoan taxa, illustrated here with example species using silhouettes identified in Fig. 2. Pathogen response: The activation of NF-κB in response to pathogens likely results in an immune response and pathogen elimination (red circle). Differences in baseline NF-κB expression may explain differential disease susceptibility (green check vs. red circle) between individuals or species. Stress response: Environmental stressors (red lightning bolt) can induce upregulation of NF-κB, which may help organisms survive stress (green check). An increase in the expression of NF-κB following exposure to stress (red lightning bolt) may promote stress tolerance (green symbol) upon repeated stress exposure. Symbiosis: The establishment of symbiosis often involves the downregulation of host NF-κB to tolerate the symbiont (brown). The upregulation of NF-κB following exposure to stress (red lightning bolt) may result in the loss of symbionts. Development: The expression of NF-κB is variable over development in some species (black triangle). Evidence from several species suggests a role of NF-κB in the differentiation of neuron-related cell types, including neurons (top black symbol) and cnidocytes (bottom black symbol).

As described in this review, there is growing knowledge of the diversity of NF-κB structures and biological functions across holozoans. In Fig. 5, we summarize known and proposed roles of NF-κB in various holozoan biological processes. These findings have led to numerous open questions related to our understanding of the varied roles of this important TF across evolutionarily distant phyla. Based on current knowledge, we outline some of these questions and suggest directions for future research.

The conserved structures and posttranslational regulation of NF-κB in vertebrates by proteolysis of ANK repeat sequences and cytoplasmic-nuclear shuttling have been known in great detail for some time (eg Fig. 1). Interestingly, although holozoan NF-κBs have broad structural diversity, findings from a diverse set of species suggest a conserved role for the ANK repeats in maintaining NF-κB in an inactive state in the cytoplasm, with ANK repeats being either part of the NF-κB protein itself or encoded by a separate IκB-like gene (Williams and Gilmore 2020; Williams et al. 2025). Yet, whether these NF-κB proteins are primarily regulated by posttranslational degradation of ANK repeats and/or via modulated transcription of IκB and NF-κB genes is not clear (eg see Mansfield et al. 2017; Williams et al. 2025). The structural and molecular characterization of NF-κB from a broader diversity of holozoan taxa would help address these important questions.

The modulation of expression and activity of NF-κB in a broad diversity of holozoan species as a consequence of pathogen exposure suggests a role in innate immunity, congruent with the broad role that this protein plays in insect and vertebrate immunity. Moreover, the differential expression of NF-κB and/or upstream signaling components under various biotic and abiotic stressors (eg heat and starvation) has also been demonstrated in several holozoan species, yet downstream cellular and physiological outcomes of this activation remain largely uncharacterized. In the case of the coral–dinoflagellate mutualism, it is now known that NF-κB activity is increased in response to heat stress, which also induces the breakdown of symbiosis (ie coral bleaching), suggesting a link between these phenomena. Exploring these hypotheses could inform conservation efforts as many marine invertebrates face collapse due to changing environmental conditions and new pathogen outbreaks. That being said, findings from the model cnidarian *E. diaphana* demonstrate the complex and unresolved relationship between NF-κB expression, stress, and pathogen susceptibility, as starved anemones both with and without symbionts displayed increased NF-κB, which was associated with increased pathogen susceptibility for aposymbiotic animals but decreased susceptibility for symbiotic animals (Valadez-Ingersoll et al. 2024). As such, additional research is needed to clarify how NF-κB influences pathogen and stressor susceptibility in multistressor scenarios.

In a diverse set of taxa including *C. owczarzaki*, *A. queenslandica*, *C. hemisphaerica*, and *N. vectensis*, NF-κB also appears to play an important role in developmental processes, which is consistent with findings on NF-κB from *D. melanogaster* (Espín-Palazón and Traver 2016). Further investigation in this area could provide foundational knowledge of the regulation of animal development. Specifically, the application of cutting-edge technologies developed to study protein–DNA interactions (eg chromatin immunoprecipitation), single-cell RNA expression profiling, and manipulative functional studies—including gene knockouts or gene knockdowns of NF-κB using techniques such as RNA interference and CRISPR/Cas9—are needed to characterize the varied functions of NF-κB and its target genes in the biological processes discussed herein. Overall, efforts to further elucidate the roles of NF-κB in the biology of phylogenetically diverse holozoan taxa will continue to be important in understanding basic and applied biology across systems and disciplines.

## Data Availability

All original data and code can be found in a public, permanent Dryad repository at https://doi.org/10.5061/dryad.t1g1jwtfx.
